# PubNet: a flexible system for visualizing literature derived networks

**DOI:** 10.1186/gb-2005-6-9-r80

**Published:** 2005-08-16

**Authors:** Shawn M Douglas, Gaetano T Montelione, Mark Gerstein

**Affiliations:** 1 Department of Molecular Biophysics and Biochemistry, Yale University, New Haven, CT 06520, USA; 2 Department of Molecular Biology and Biochemistry, Center for Advanced Biotechnology and Medicine, Rutgers University and Robert Wood Johnson Medical School, Piscataway, NJ 08854, USA; 3 Department of Computer Science, Yale University, New Haven, CT 06520, USA

## Abstract

We have developed PubNet, a web-based tool that extracts several types of relationships returned by PubMed queries and maps them into networks, allowing for graphical visualization, textual navigation, and topological analysis. PubNet supports the creation of complex networks derived from the contents of individual citations, such as genes, proteins, Protein Data Bank (PDB) IDs, Medical Subject Headings (MeSH) terms, and authors. This feature allows one to, for example, examine a literature derived network of genes based on functional similarity.

## Abstract 2

PubNet is a web-based tool to extract several types of relationships returned by PubMed queries and map them into networks.

## Rationale

The amount of widely accessible scientific data has increased dramatically in recent years. There are currently more than 31,000 structures in the Protein Data Bank (PDB) [1], as compared with 3,000 structures 10 years ago. Swiss-Prot [2] now contains more than 178,000 sequence entries, which is up from 40,000 in 1994. With continual advances and refinements of experimental and computational technologies, data creation promises to accelerate for the foreseeable future.

PubMed [3] stands out as a key information resource in the biological sciences in terms of diversity, breadth, and manual curation. PubMed entries comprise an order of magnitude more data than the three billion bases of the human genome. In addition to basic citation and abstract information, PubMed provides rich meta-information including Medical Subject Headings (MeSH) terms, detailed affiliation, and any secondary source databanks and accession numbers of molecules discussed in each article. By parsing the XML output of a query and performing a few simple operations, it is possible to uncover many interesting relationships among publications.

Previous work has been done to augment or refine the standard PubMed search, including tools to conduct combinatorial searches [4] and to navigate standard search results based on common MeSH terms [5], gene names found in abstracts [6,7], PubMed-assigned 'related articles' [8], and combinations thereof [9-12]. In PubNet we present a unique two-pronged approach in which network graphs are dynamically rendered to provide an intuitive and complete view of search results, while hyperlinking to a textual representation to allow detailed exploration of a point of interest. Multiple simultaneous queries are also supported, greatly increasing the number and types of relationships that can be visualized. The PubNet server, source code, and gallery are available on the worldwide web [13].

## How PubNet works and interpreting the output

**Figure 1:**
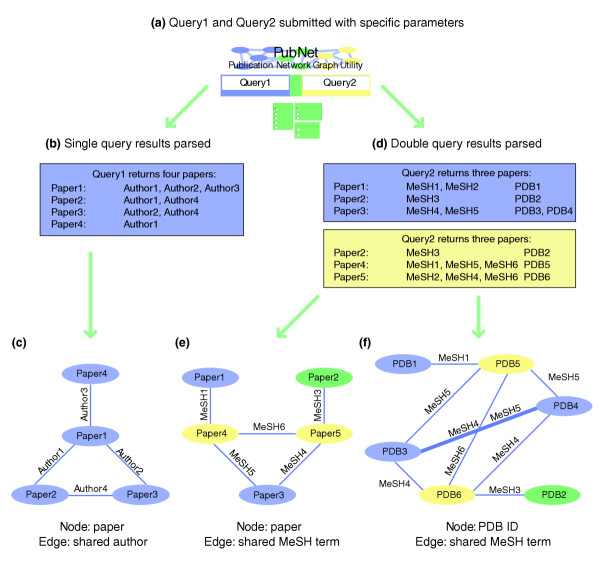
Basic examples. **(a) **The main page allows for submission of one or two queries. Queries are entered into the blue and yellow text boxes, and parameter options are selected below. Nodes may be defined as author, paper, Protein Data Bank (PDB), Genbank, or Swiss-Prot ID, and edges may be drawn for co-authorship, shared Medical Subject Headings (MeSH) term, or shared location. **(b) **PubNet connects to PubMed, submits each query separately, and parses the XML results. In this example, only Query1 was submitted, returning four publications. **(c) **In the output, each paper is represented as a single node. Each pair of nodes that share a common author are linked by an edge. **(d) **In this example, Query1 and Query2 have each returned three papers, each with MeSH terms and PDB IDs. **(e) **When nodes are specified as papers and edges specified as shared MeSH terms, papers returned only by Query1 are represented as blue nodes, papers returned only by Query2 are shown in yellow, and papers common to both queries are shown in green. **(f) **When nodes are specified as PDB IDs and edges specified as shared MeSH terms, each PDB ID from each paper is represented as a node and colored according to the query from which it was derived. A single paper can give rise to multiple nodes, as is the case for Paper3, which contains two PDB identifiers, each of which are represented by a separate node.

Visualizing a publication extracted network is done by entering at least one PubMed query into the provided textbox, selecting node and edge parameters, and clicking 'Submit' (Figure 1a). Each query is relayed to PubMed, and so all standard PubMed syntactical conventions apply. The PubMed XML output is parsed and the network graphs are drawn with the aid of aiSee graph visualization software [14]. The simplest PubNet example is the network relating papers by shared authorship, generated from a single query (Figure 1b and 1c). In this example, there is a one-to-one correspondence between the number of papers returned by the query and the number of nodes drawn on the graph. Each pair of papers is then linked by an edge if they share at least one common author, and edges are scaled in thickness for multiple common authors. Much more complex networks can be derived by entering two queries and selecting node parameters for which there may be a one-to-many correspondence between papers returned by PubMed and nodes associated with each paper (Figure 1d-f). As is often the case when nodes are set to Author or Databank ID, each publication returned by each query will expand to several nodes in the final network display. Nodes are colored according to the query from which they are derived, allowing for greater information content than would an otherwise identical monochrome graph. For example, the degree to which nodes of different colors segregate or overlap can suggest specific relationships between the publications in the query results.

**Figure 2:**
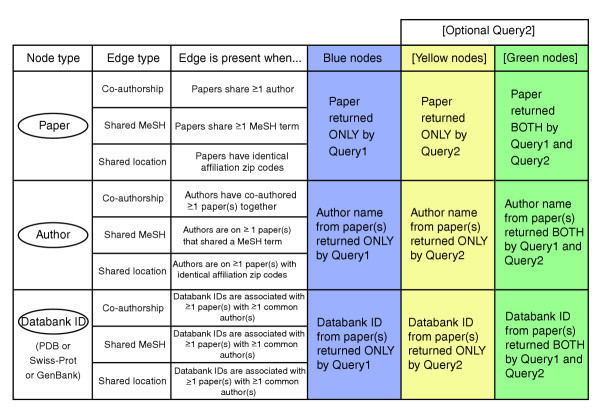
Node and edge reference chart. Nodes and edges can have subtle meanings depending on the parameters used to draw graphs with PubNet. This chart can be used as an aid when interpreting complex graphs. MeSH, Medical Subject Headings; PDB, Protein Data Bank.

The graphical representation of a network is meant to provide a broad overview of the structure of meta-relationships returned by one or two queries. Each graph is downloadable in a variety of formats, including SVG, PS, PDF, and PNG. The vector formats permit image rescaling without loss of quality. Depending on the input queries and parameters, the specific coloring and arrangement of nodes and edges can mean a variety of different things. In all cases, nodes that were derived from the first query are colored blue, nodes derived from the second query are colored yellow, and nodes derived from papers appearing in both queries are colored green. Figure 2 can be used as a reference for interpreting the meaning of nodes and edges for each of the parameter combinations.

Generally speaking, subsets of nodes that are highly connected are drawn together in tight clusters, whereas sparsely connected nodes are spread further apart. If two queries are entered, then the degree to which the two colors overlap on the graph can also be significant. These relationships can be compared quantitatively by exporting the network to TopNet [15], which calculates average degree, clustering coefficient, characteristic path length, and diameter for any network. TopNet automatically scores PubNet networks by clicking the 'Export to TopNet' icon below any PubNet query result.

Hyperlinks to a textual representation of every graph are provided on its results page. The textual representation provides a summary list of all nodes and edges that comprise the network. Each entry in the summary is a hyperlink to a detailed description. For nodes, a list of outgoing edges as well as a list of all connected neighbors and their respective edges are shown, with common edges highlighted. Relevant external databank links are also provided at the top of the page. The detailed view of an edge shows a list of all nodes connected by that edge. Note that in the SVG graphical format each node is also a hyperlink to its entry in the text version of the network, which allows one to navigate quickly from an interesting region in the graph to a detailed description of its components.

## Applications

**Figure 3:**
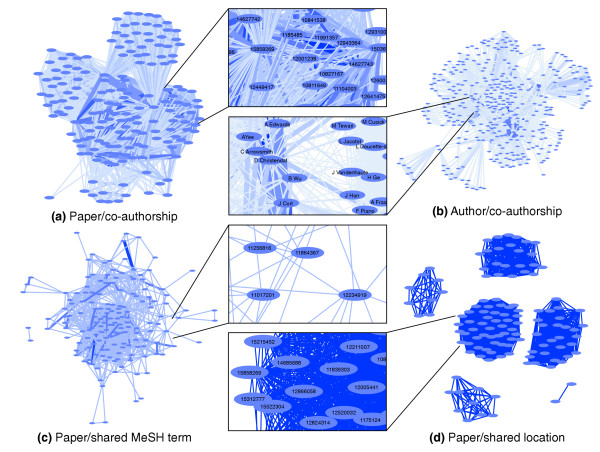
Collaborative organization of the Northeast Structural Genomics (NESG) consortium. **(a) **Paper nodes linked by co-authorship edges show four major groups of publications, roughly corresponding to individual laboratories from which they were published. Here, three of the groups are fairly well connected to form the central nodes in the graph. The fourth set of papers is internally well connected but is only linked to other groups by a single paper with shared authors. **(b) **When nodes are drawn as authors with co-authorship edges, a slightly different pattern emerges. The principal investigators from each laboratory tend to form central anchor points, from which other laboratory members branch out. Links also connect collaborating laboratories. **(c) **Yet another pattern arises when shared Medical Subject Headings (MeSH) terms are used to connect papers. As expected, a large and well connected group of nodes is drawn in the center. Several unconnected nodes lining the periphery show papers that are unrelated in subject matter to the main group. **(d) **When papers are linked by shared zip codes, large clusters arise corresponding to geographically disparate laboratories. Here, the main clusters are the Universities of Washington, Columbia, Yale, Buffalo, and Rutgers.

Recent advances in high throughput techniques have made it possible to conduct biomedical research on a larger scale than was previously possible. These efforts often involve large groups of scientists from multiple institutions working in close collaboration on high throughput experiments, data collection, and analysis. There is little precedent in the biological sciences for executing or evaluating such large scale endeavors, but in the latter case a logical place to start is the product of those endeavors, namely publications. As we demonstrate below, the organization and output of a collaboration is very well reflected by patterns that can be extracted from its publication list in Figure 3.

**Figure 4:**
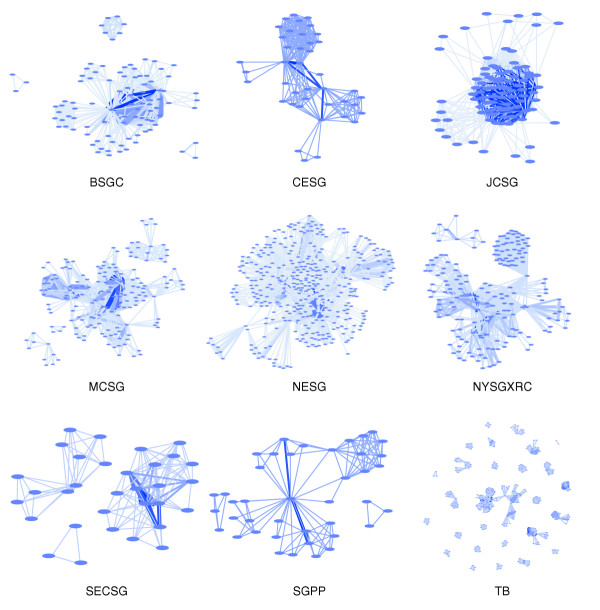
Author/co-authorship graphs for nine pilot centers of the Protein Structure Initiative. Publications were collected from the official publication lists of the following centers: Berkeley Center for Structural Genomics (BSGC), Center for Eukaroytic Structural Genomics (CESG), Joint Center for Structural Genomics (JCSG), Midwest Consortium for Structural Genomics (MCSG), Northeast Structural Genomics (NESG) consortium, New York Structural Genomics Research Consortium (NYSGXRC), Southeast Consortium for Structural Genomics (SECSG), Structural Genomics of Pathogenic Protozoa (SGPP), and Tuberculosis Structural Genomics (TB) consortium. The MCSG, NESG, and NYSGXRC consortia have comparably greater authorship of publications, with individual laboratories clustering together. The BSGC and JCSG centers include nearly all participating authors on every publication, as seen by dense clusters with heavy edge weights. At the other extreme, the TB consortium is a loose collaboration of many groups of different scientists who tend to publish separately.

The Protein Structure Initiative (PSI) is a large-scale effort led by the US National Institutes of Health that is aimed at streamlining the process of three-dimensional protein structure determination, with the long range goal of providing three-dimensional structures of most proteins in nature. Nine structural genomics research centers are supported by the PSI, each of which has its own expertise, organization, and research focus [16]. To demonstrate the versatility of PubNet, we generated several graphs based on publication lists from each PSI center (Figure 4), including the Northeast Structural Genomics (NESG) consortium.

Structural genomics centers attempt to solve structures at very high throughput, and each center has its own unique approach to accomplish this task. Because the PSI is still in its pilot stages, it is yet to be determined which approach is the most successful. Here we show how organizational, geographic, and social patterns of large collaborative research efforts are reflected in their publications.

### Collaborative organization of single consortium

We begin by illustrating the types of relationships that can be extracted from a single query (Figure 3). A query consisting of a list of all NESG PubMed IDs was analyzed using four different combinations of node and edge types, and each yielded strikingly different graph structures. Depending on the parameters that were specified to generate the graph, these linkages may correspond to similarity between papers, frequency of copublication between two authors (for a given query), common geographic sources for publications, and so on. The scalable vector graphics formats supported by PubNet allow one to zoom in on specific regions in the graph. Each node in the graph image is hyperlinked to a detailed textual report, which includes a hyperlinked list of all outgoing edges and a list of all neighboring nodes with their respective edges. Thus, starting directly from the graphical output, it is possible to explore specific node-edge linkages in detail.

In the graph shown for the NESG consortium in Figure 3b, nodes are authors (researchers) and edges represent co-authorships on publications. It demonstrates the confederated but coordinated approach used by the NESG consortium, which includes two protein sample production centers, at least six different sites at which three-dimensional structures are determined by nuclear magnetic resonance or X-ray crystallography, and a loosely coupled group of some dozen laboratories working on various aspects of the technology development and annotation.

### Comparison of several consortia

We also compare the publication authorship patterns of each of the PSI centers in Figure 4, using nodes to represent authors and edges to represent co-authorship. Because a single set of parameters was used across multiple queries, the underlying relationships between nodes are identical for each graph, and so differing graph structures correspond to variations in the global structure of these relationships. A diverse array of graph structures is evident, highlighting significant differences in size, frequency in publication, and degree of cooperation across the consortia. For example, the Tuberculosis Structural Genomics consortium [17] conducts its experiments in small separate groups, whereas the Joint Center for Structural Genomics [18] uses a more centralized approach. Groups such as the NESG [19] and New York Structural Genomics Research Consortium [20] employ an intermediate approach, in which central groups are tightly clustered but also linked to other groups in a collaborative pipeline.

## A simple example with Protein Data Bank IDs

**Figure 5:**
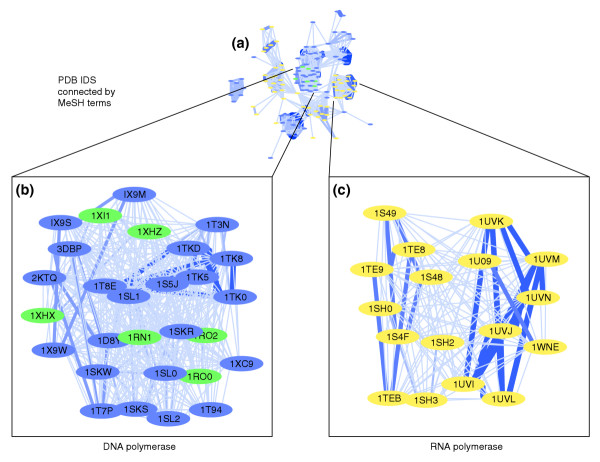
DNA and RNA polymerase Protein Data Bank (PDB) IDs connected by Medical Subject Headings (MeSH) terms. **(a) **The PubNet graph resulting from the queries 'DNA polymerase 2004[dp]' and 'RNA polymerase 2004[dp]'. **(b) **A magnified view showing several PDB IDs that were present in papers returned by the first query. PDB IDs that were only returned by the 'DNA' query are blue, and those returned by both queries are shown in green. The green nodes correspond to structures of DNA primase. **(c) **A magnified view of several RNA polymerase PDB IDs.

In addition to extracting and rendering authors and papers as nodes, PubNet is able to use databank accession numbers found in PubMed citations, such as PDB, GenBank, or Swiss-Prot IDs. These databanks have tens or hundreds of thousands of entries, and so when using databank IDs as nodes it is often useful to limit the scope and date range of queries to PubNet to avoid overly complex results. Figure 5 shows a basic example using PDB IDs as nodes and MeSH terms as edges. The first query, namely 'DNA polymerase 2004[dp]', is limited to a specific type of protein and to papers published in 2004. The second query - 'RNA polymerase 2004[dp]' - is similar. Blue nodes cluster tightly together, as do yellow nodes, indicating that they are highly similar. Nodes in separate clusters are connected to each other in some cases. By examining the textual view of the nodes, it is easy to understand the underlying structure. Predictably, blue nodes are highly linked to each other by MeSH terms related to DNA polymerase, such as 'DNA-directed DNA polymerase'. Yellow nodes are linked by terms such as 'RNA polymerase II'. Blue and yellow nodes occasionally link to each other by terms such as 'Models, molecular'. Green nodes, which are nodes that were extracted from papers returned by both queries, are linked to each other by the term 'DNA primase' and to other blue nodes by 'DNA-directed DNA polymerase'.

## Evaluating the output of the Protein Structure Initiative

**Figure 6:**
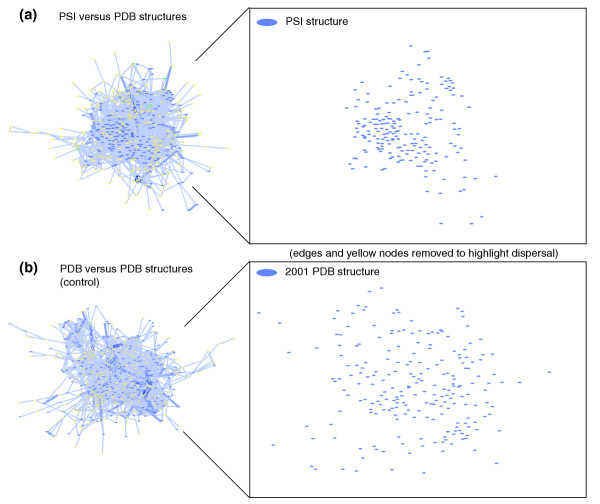
Structure publications linked by Medical Subject Headings (MeSH) terms. **(a) **All available primary citations for Protein Structure Initiative (PSI) structures (shown in blue) were compared with primary citations from a random set of 300 Protein Data Bank (PDB) structures (shown in yellow). Blue color nodes are segregated into tight clusters, indicating close similarity among PSI structures. Yellow nodes are relatively interspersed because of fewer common edges, indicating greater variability in connecting MeSH terms. Out of a total of 860 MeSH terms associated with PSI structure nodes (435 of which are unique), the term 'Bacterial proteins - chemistry' is the most common, connecting a clique of 52 nodes. **(b) **As a control, two random sets of 300 PDB structures were chosen from a set of 3,112 structures released in 2001-2002 that included a primary citation available in PubMed. Those sets of citations were then run through PubNet as Query1 and Query2. Nodes of both colors are fairly well interspersed. The PDB structure nodes carry 1,247 MeSH terms (861 unique), the most common of which is also 'Bacterial proteins - chemistry', but it only connects a clique of 16 nodes.

Figure 5 is an illustrative example; we present Figure 6 as a more practical example of the use of PubNet. To investigate the extent to which PSI structures are representative of all PDB structures, we compared several two-query PubNet graphs based on PSI and non-PSI structure publications. Two representative graphs are shown in Figure 6. To construct the queries, lists of primary citation PubMed IDs were compiled using the PDB search engine. The structural genomics PDB IDs were extracted from TargetDB [21], and sets of 300 regular PDB IDs were selected randomly from a total of 3,112 unique structures released in 2001-2002 that included a primary citation available in PubMed. Nodes were designated as papers and edges as shared MeSH terms. Because only primary citations were used, there is a one-to-one mapping of papers to PDB structures. Each node thus corresponds to a PDB structure, and the associated MeSH terms provide a description of that structure. Functional similarity among a subset of structures results in more common MeSH terms, which is reflected in the graph by greater connectivity of the nodes, and tighter clustering of the nodes relative to dissimilar nodes on the graph.

To compare PSI structures with general PDB structures, two types of graphs were generated. First, a two-query graph was generated with all available PSI structure associated PubMed IDs comprising the first query, and a random set of 300 PDB IDs comprising the second query (Figure 6a). The second type of graph was generated by running two random sets of 300 PDB IDs against each other (Figure 6b).

We have observed that differing patterns in PubNet graphs among ostensibly similar queries can reveal underlying differences derived from the content of the publications returned by each query. Major features that can vary include the degree of aggregation of nodes into different clusters (roughly indicating the subject of the protein structure) and the balance of both blue and yellow nodes within the various clusters. If PSI structure publications are indistinguishable from random PDB structure publications, then we would expect the graphs based on PSI structures publications versus random PDB structure publications to have a similar character to graphs based on two random sets of PDB structures. However, as shown in Figure 6a, the PSI structure publication nodes do not intersperse with regular PDB structure nodes as much as two sets of random structures. The PSI nodes clearly tend to aggregate in tighter neighborhoods than do the other nodes. Although this is by no means definitive, the differential clustering might indicate some underlying differences between the PSI structures and random PDB structures. One obvious source of difference in the structure publications is the fact that many PSI structures are un-annotated 'hypothetical proteins', and so they lack the MeSH terms required for greater dispersal. Another factor might be that similar methods are used to determine PSI structures, and this is reflected in their publications.

## Assessing results with TopNet

In addition to examining the textual representation of the graph, qualitative assessments of the network structure can be verified by exporting the results of any PubNet query to TopNet. One particularly useful descriptor is the average degree of a network, which is the average of the degrees of each node. In a PubNet graph, node degrees increase with more common edge terms between the nodes. A high average degree indicates that the nodes are highly connected to each other. Note that the utility of many topological descriptors depends on the connectedness of a graph. For a more detailed explanation of descriptors, see the report by Yu and coworkers [15].

In Table 1, we compare the TopNet generated graph statistics of several graphs shown in figures cited above. In Figure 4 the Joint Center for Structural Genomics graph is highly connected, and the Tuberculosis Structural Genomics consortium graph is sparsely connected. This difference is particularly evident in the 'average degree' scores for each graph. In Figure 5 we see that nodes from the two 'polymerase' queries are very similar in layout and connectedness. As expected, their TopNet scores are nearly identical. For Figure 6 we see that PSI nodes have a much higher average degree, lower diameter and average distance, and increased clustering coefficient when compared with random sets of PDB nodes. We note that when looking at a large numbers of nodes, even small differences in graph statistics are meaningful. Each feature of the graph confirms what is clearly visible in the graphical output; PSI nodes are better connected to each other and cluster more tightly together in comparison with random PDB nodes.

## Conclusion

In this paper we present PubNet, a web tool that can be used to extract and visualize a variety of relationships between publications indexed by PubMed. Distinguishing features of PubNet include its ability to generate several different types of graphs based on a single query and to accommodate two queries simultaneously, which greatly facilitates graph comparison. The basic functionality of PubNet is demonstrated by its application to publications derived from the PSI, which revealed a diverse array of collaborative patterns in the different research centers as well as increased similarity between primary citations associated with those structures relative to a random sample of PDB structure citations. It is unclear whether, once properly annotated, these differences will remain.

By focusing on PSI publications we offer only a small glimpse of the possible uses of PubNet. Although only 15 combinations of node and edge parameters are currently supported, the number of different queries that can be entered is unrestricted. We have included a 'save' feature that permanently links any PubNet graph to a user gallery, and we invite the community to submit queries and comments.

## Figures and Tables

**Table 1:** TopNet comparison of several networks

Source	Average degree	Average distance	Clustering coefficient	Diameter
Figure 4 (NESG IDs)	19	2.7	0.43	6
Figure 4 (JCSG IDs)	45	1.5	0.46	3
Figure 4 (TB IDs)	6.7	2.0	0.46	4
Figure 5a (DNA pol IDs)	15	3.1	0.44	7
Figure 5b (RNA pol IDs)	13	3.1	0.44	7
Figure 6a (PSI IDs)	24	2.6	0.37	7
Figure 6b (PDB IDs 1)	6.1	3.9	0.31	9
Figure 6b (PDB IDs 2)	6.7	3.8	0.33	10

JCSG, Joint Center for Structural Genomics; NESG, Northeast Structural Genomics; PDB, Protein Data Bank; pol, polymerase; TB, Tuberculosis Structural Genomics.
